# Integrated Proteomic and Transcriptomic Analysis of Gonads Reveal Disruption of Germ Cell Proliferation and Division, and Energy Storage in Glycogen in Sterile Triploid Pacific Oysters (*Crassostrea gigas*)

**DOI:** 10.3390/cells10102668

**Published:** 2021-10-05

**Authors:** Chen Chen, Hong Yu, Qi Li

**Affiliations:** 1 Key Laboratory of Mariculture, Ministry of Education, Ocean University of China, Qingdao 266003, China; chenchen920@stu.ouc.edu.cn (C.C.); qili66@ouc.edu.cn (Q.L.); 2 Laboratory for Marine Fisheries Science and Food Production Processes, Qingdao National Laboratory for Marine Science and Technology, Qingdao 266237, China; 3 Laboratory of Tropical Marine Germplasm Resources and Breeding Engineering, Sanya Oceanographic Institution, Ocean University of China, Sanya 572000, China

**Keywords:** *Crassostrea gigas*, proteome, transcriptome, sterility, triploid

## Abstract

Triploid oysters have poor gonadal development, which can not only bring higher economic benefits but also have a potential application in the genetic containment of aquaculture. However, the key factors that influence germ cell development in triploid oysters remain unclear. In this study, data-independent acquisition coupled to transcriptomics was applied to identify genes/proteins related to sterility in triploid *Crassostrea gigas*. Eighty-four genes were differentially expressed at both the protein and mRNA levels between fertile and sterile females. For male oysters, 207 genes were differentially expressed in the transcriptomic and proteomic analysis. A large proportion of downregulated genes were related to cell division, which may hinder germ cell proliferation and cause apoptosis. In sterile triploid females, a primary cause of sterility may be downregulation in the expression levels of certain mitotic cell cycle-related genes. In sterile triploid males, downregulation of genes related to cell cycle and sperm motility indicated that the disruption of mitosis or meiosis and flagella defects may be linked with the blocking of spermatogenesis. Additionally, the genes upregulated in sterile oysters were mainly associated with the biosynthesis of glycogen and fat, suggesting that sterility in triploids stimulates the synthesis of glycogen and energy conservation in gonad tissue.

## 1. Introduction

Polyploidy, or whole-genome duplication, is a frequent phenomenon in animals [1]. Triploids possess three sets of chromosomes in somatic cells instead of two in normal diploids. The odd number of homologous chromosomes may cause unequal separation during meiosis, resulting in retarded gonadal development or aneuploid gametes. Triploids can occur across all major animal groups but are often lethal in some vertebrates, such as birds and mammals. It is easy to cause dead embryos or lethal abnormalities [2,3], which makes triploids are less common in the wild, whereas in fishes, amphibians, and lizards [4,5,6], triploids may develop and display relatively normal phenotypes. However, some species have large sizes with faster growth compared to normal diploids. In many aquaculture organisms, such as fishes, shrimps, and shellfishes, triploids are viable but tend to be sterile due to a lack of gonadal development. The sterility can allow for faster growth rates as the energy invested in reproduction can be diverted to somatic growth [7,8]. Therefore, triploid induction has gained much attention due to its great commercial benefits in aquaculture. Besides the high potential for commercial application, triploids also have significant potential applications in the genetic containment of aquaculture species [9].

The Pacific oyster, *Crassostrea gigas* is of great ecological and economic importance, providing important ecological services as filter-feeders and reef-builders [10,11], and contributing weightily to the aquaculture industry worldwide [12]. Typically, they are diploid organisms characterized by the presence of complete paternal and maternal genomes in each somatic cell, but triploids can occur spontaneously at a low but steady frequency in natural populations of *C. gigas* [13]. During the spawning season, the majority of a diploid Pacific oyster’s energy is directed toward reproduction, and flesh palatability decreases as gonad replaced stored glycogen [14]. However, triploid oysters are always sweet and have the desired texture due to their sterility and reduced gonad development. There are two major techniques to produce triploid oysters: via chemical induction or through mating a diploid and a tetraploid. Originally, triploid oysters were produced by inhibiting polar body formation in newly fertilized eggs with cytochalasin B [15]. However, the use of cytochalasin B in triploid induction has been criticized due to its toxicity and inefficiency. Nowadays, triploid oysters are produced mainly by mating female diploids and male tetraploids [16]. Triploid oysters have become a popular commercial product due to their faster growth [17,18,19], greater disease resistance [20], and improved meat quality [21].

The sterility trait is important for oyster breeding since oysters use more than 50% of their tissue weight for gonad production [22]. The important value of triploid oysters is principally due to their reduced fecundity. However, triploid oysters are not 100% sterile and a proportion do produce some gametes. Hence, gonad development in triploid oysters can be roughly divided into two types: partial fertility and sterility. As studied by Jouaux et al. [23], some triploid Pacific oysters showed advanced gametogenesis (named 3nα), while some triploids were almost sterile with a small number of gametes (named 3nβ) at stage Ⅲ. In *C. virginica*, triploids were classified according to the types of gonia present (α gonia or β gonia). α gonia (“normal” gonia) were usually associated with males, whereas β gonia (irregular gonia) suggested female lineages. Sterile types included inactive triploids that had no oocytes or spermatogenic cells, oligo females of which none or a few oocytes were observed among all follicles, and virilescent females of which follicles were lined with β gonia but contain spermatozoa [24]. The complicated fecundity of triploid oysters has attracted the attention of researchers. In summary, the main manifestation of sterile triploid oysters is a significant reduction in gamete production. Hence, understanding the mechanisms regulating gametogenesis in triploids is an important area of interest.

The Pacific oysters constitute a unique model for investigating the germ cell development of triploids. Some studies have been conducted on triploid gametogenesis and gonadal development of *C. gigas* such as by histology, gamete quality [25], and transcriptomic analysis [26]. However, the mechanisms regulating gametogenesis in triploid Pacific oysters are far from being fully elucidated and require further in-depth studies.

Proteins are the direct functional performers of an organism and control critical organismal phenotypes, so the research on their expression levels has an irreplaceable advantage. Fast-forward to today’s exciting era of high throughput sequencing enables large-scale proteomic profiling, which has opened up new perspectives in every field of biology. Proteomic approaches integrated with transcriptomics have been applied successfully to reveal the molecular mechanisms involved in reproduction and development in numerous animals [27,28,29,30].

Accordingly, to promote our understanding of the gonadal sterility in triploid oysters, a comparative proteomic analysis, integrated with transcriptomics was conducted in this study. The findings provide new insights into the underlying molecular mechanisms of governing the differences between fertile oysters and sterile triploid oysters.

## 2. Materials and Methods

### 2.1. Ethics Statement

The pacific oyster is neither an endangered nor protected species. All experiments in this study were conducted according to national and institutional guidelines.

### 2.2. Sample Collection, Ploidy Verification, and Histology

Two-year-old triploid and diploid oysters were collected from Weihai, Shandong province, China, in late June 2018. The triploids had been produced by mating tetraploid males and diploid females, and ploidy was verified via Cyto-FLEX flow cytometer (Beckman Coulter, Brea, CA, USA) using DAPI (Sigma–Aldrich, St Louis, MO, USA) as a stain. Gill filament tissues were obtained and prepared into cell suspension in 1 mL PBS (Phosphate Buffer Saline). Then, DAPI (final concentration: 0.6 µg/mL) was added to the cell suspension and the ploidy was detected after filtration with 300 mesh sieve silk. Diploid cells were used as the control, and the peak values of triploids were 1.5 times higher than that of diploids. Gonad tissue of each individual was sampled either frozen in liquid nitrogen immediately, and then stored at −80 ℃ until use, or fixed in Bouin’s solution for histological analysis.

### 2.3. RNA Extraction, Transcriptome Sequencing and Analysis

Three individual gonad samples for each sex of diploid oysters, each sex of fertile triploids, and each sex of sterile triploids were prepared for transcriptomics analysis. Samples were named as follows: male diploids (M-2n-1, M-2n-2, M-2n-3), female diploids (F-2n-1, F-2n-2, F-2n-3), male fertile triploids (M-3nα-1, M-3nα-2, M-3nα-3), female fertile triploids (F-3nα-1, F-3nα-2, F-3nα-3), male sterile triploids (M-3nβ-1, M-3nβ-2, M-3nβ-3) and female sterile triploids (F-3nβ-1, F-3nβ-2, F-3nβ-3). Total RNA of gonad tissues was extracted using TRIzol reagent (Invitrogen, Carlsbad, CA, USA) according to the manufacturer’s protocol. RNA concentration and purity were assayed using Nano Drop 2000(Thermo Fisher Scientific, Waltham, MA, USA), and the 1% agarose gel electrophoresis was performed to confirm the quality of RNA. The RNA integrity number (RIN) was assessed using the Agilent 2100 Bioanalyzer (Agilent Technologies, Palo Alto, CA, USA). RIN ≥ 7 was set as the cutoff for RNA quality. Totally, eighteen different libraries were constructed. The libraries were sequenced on Illumina HiSeq™ 2500 sequencing platform and 150 bp paired-end reads were generated. All raw data were submitted to the NCBI sequence read archive (SRA) database under accession number PRJNA690125.

Bowtie2 was used to map the reads against the ribosome RNA (rRNA) database to delete reads mapped by rRNA. The RNA-seq reads were mapped onto the *C. gigas* genome (unpublished) using the default TopHat 2 v2.0.3.12 package. RSEM v1.2.19 package was used to quantify the abundance of each transcript, and the FPKM was applied to normalize the transcript expression levels. Differentially expressed genes (DEGs) between different groups were identified by DESeq2 v1.4.0 software (Bioconductor, Seattle, WA, USA) using the following filter criteria: |log2FC| > 1 and FDR < 0.05 [31].

### 2.4. Proteome Sequencing and Analysis

#### 2.4.1. Protein Extraction and Protein Digestion

Eighteen libraries were constructed for proteomic analysis. Total proteins were extracted separately from the same samples as those for RNA-seq using the cold acetone method. The Pierce™ BCA Protein Assay Kit (Thermo Fisher Scientific, Waltham, MA, USA) was used to determine the protein concentration of the supernatant. Proteins were then digested quickly and entirely into peptides with sequence-grade modified trypsin (Promega, Madison, WI, USA).

#### 2.4.2. Generation of the Spectral Library

All sample peptide mixtures were dissolved and then fractionated by high pH separation using Ultimate 3000 system (Thermo Fisher Scientific, Waltham, MA, USA) connected to a reverse phase column (XBridge C18 column, 4.6 mm x 250 mm, 5 μm; Waters Corporation, Milford, MA, USA). High pH separation was performed using a linear gradient. The column was re-equilibrated for 15 min under initial conditions. Six fractions were collected and each fraction was dried in a vacuum concentrator to be used.

The desalted lyophilized peptides were redissolved and analyzed by LC-MS/MS equipped with an online nanojet ion source. The complete system was an Orbitrap Lumos mass spectrometer in tandem with an EASY-nLC 1200 system (Thermo Fisher Scientific, Waltham, MA, USA).

Raw data of data-dependent acquisition (DDA) were processed and analyzed by Spectronaut X (Biognosys AG, Schlieren, Switzerland) with default settings to generate an initial target list. The FDR was set to 1% for both parent ion and peptide levels.

#### 2.4.3. Mass Spectrometry Data Analysis

Raw data of data-independent acquisition (DIA) was processed and analyzed by Spectronaut X (Biognosys AG, Schlieren, Switzerland) with default parameters. Spectronaut Pulsar X will determine the ideal extraction window dynamically depending on iRT calibration and gradient stability. FDR (q-value) cutoff on precursor and protein level was applied 1%. The average top three filtered peptides which passed the 1% FDR cutoff were used to calculate the major group quantities. After Student’s *t*-test, different expressed proteins were filtered if their q-value < 0.05 and Absolute AVG log2 ratio > 0.58. In addition, we identified proteins with |log2FC| > 0.58 and q-value < 0.05 between different groups as significant differentially expressed proteins (DEPs).

All raw mass spectrometry data were deposited in the Integrated Proteome Resources (iProX; http://www.iprox.org, accessed on 23 August 2021) database under the accession number IPX0003407000.

#### 2.4.4. Bioinformatics Analysis

Based on the expression results of each sample, principal component analysis (PCA) was performed using the R software package to investigate sample repeatability.

Correlation analysis of transcriptome and proteome was performed by R software package. Nine-quadrant maps were drawn based on changes in the expression of the gene in the transcriptome and proteome. Quantitative analysis and enrichment analysis of genes in each region of the nine-quadrant map. The data for the identified DEGs and DEPs of gonad were submitted to Gene Ontology (GO) Terms (http://geneontology.org/, accessed on 23 August 2021) for GO analysis, by which the DEGs consistent with DEPs were assigned into three branches of ontology-cellular component, molecular function, and biological process. The Kyoto Encyclopedia of Genes and Genomes (KEGG; http://www.genome.jp/kegg/, accessed on 23 August 2021) pathway analysis was performed to reveal the potential functions of the DEGs/DEPs.

Furthermore, the protein database of clusters of euKaryotic Orthologous Groups (KOG; http://www.ncbi.nlm.nih.gov/KOG, accessed on 14 April 2020) was also used to assign possible functions to the identified proteiens. EuKaryotic Orthologous Groups were annotated with the KOG database proteins using blastp with a maximal *e*-value of e^−5^.

### 2.5. Quantitative Real-Time PCR (qRT-PCR) Analysis

We selected eleven gonadal development-related genes with the same expression trend in transcriptome and proteomics for qRT-PCR validation. Total RNAs were reversely transcribed to the first-strand cDNA using PrimeScript™ RT reagent kit with gDNA Eraser (Takara, Kusatsu, Shiga, Japan). The qRT-PCR was carried out on Light Cycler 480 instrument (Roche, Indianapolis, IN, USA) using QuantiNova SYBR Green PCR Kit (Qiagen, Hilden, Germany), according to the manufacturer’s recommendations. Five commonly used housekeeping genes, EF-1a, GAPDH, RS18, RO21, and RL7, were selected as candidate reference genes. GeNorm v3.5 software (Ghent University Hospital Ghent, Belgium) was used to evaluate their expression stabilities [32]. The primers for gonadal development-related genes were designed using Primer Premier v.5.0 software (PREMIER Biosoft International, San Francisco, CA, USA). The amplified regions of all the 11 genes selected for verification by qRT-PCR span at least one intron. Primers for candidate reference genes were derived from previous studies [33,34]. All the primers used for real-time PCR were shown in Table 1. The quantitative variation was calculated using three biological replicates with the relative quantitative method (2^−ΔΔCt^) [35]. The data from independent qRT-PCR runs were expressed as means ± standard deviation (SD). Statistical analysis was conducted by ANOVA with LSD for multiple comparisons (GraphPad Prism, version 8.0.1). Differences with a value of *p* < 0.05 were considered statistically different.

## 3. Results

### 3.1. Ploidy Verification, and Histological Observations of Gonadal Development in Diploid and Triploid Pacific Oysters

**Figure 1:**
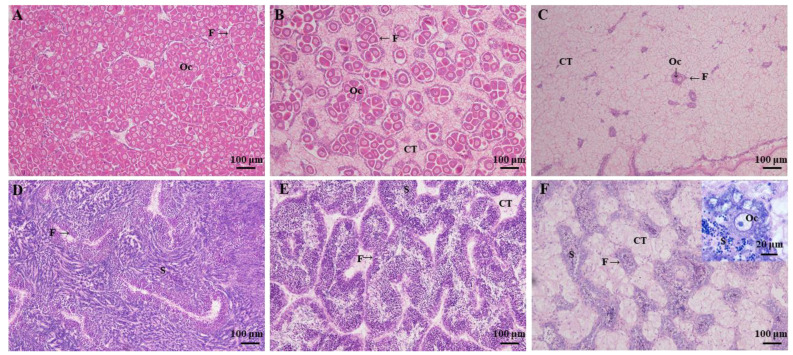
Female and male mature stages of gonad development. (**A**) Female diploids contain numerous mature oocytes. (**B**) α female triploids have a considerable number of mature oocytes. (**C**) β female triploids present a drastically reduced number of oocytes. (**D**) Male diploids are filled with mature spermatozoa. (**E**) α male triploids have large numbers of spermatozoa in their follicles. (**F**) β male triploids’ mature spermatozoa was reduced. (**G**) Male-sterile triploids were present in the form of hermaphrodites in our study. Oc: Oocyte; F: Gonadal follicle; CT: Conjunctive tissue; S: Spermatid.

Results of ploidy verification were presented in Supplementary Figure S1. Gonad development of all of the diploid oysters used in this study was at stage Ⅲ, corresponding to the stage of sexual maturation. Female follicles are filled with a large number of polygonal mature oocytes, which squeeze each other (Figure 1A); male follicles are filled with mature spermatozoa arranged in a radial pattern (Figure 1D). In triploid oysters, two patterns of maturity were observed and analyzed. For females, fertile individuals had a considerable number of mature oocytes (F-3nα) (Figure 1B); sterile animals presented a drastically reduced number of oocytes (F-3nβ) (Figure 1C). For males, fertile individuals had large numbers of spermatids and spermatozoa in their follicles (M-3nα) (Figure 1E); sterile males were present in the form of hermaphrodites, predominantly males (with few oocytes in some follicles, M-3nβ) (Figure 1F,G). No completely sterile male triploid described by Jouaux et al. [23] was observed in all the samples we collected.

### 3.2. Proteins Identified in Gonads of C. gigas

In total, 56,369 peptides were obtained and 7044 proteins were detected from the collected gonads (Supplementary Figure S2A), of which 5541 proteins were annotated and 1503 proteins were unannotated. Proteins detected with detailed information are listed in Supplementary Table S1. Among these annotated proteins, 1723 (31.10%), 3185 (57.48%), and 4920 (88.79%) were identified in the GO, KEGG, and KOG databases, respectively. 3309 (59.72%) proteins were annotated in more than one database and 978 (17.65%) proteins were simultaneously annotated in three public databases (Supplementary Figure S2B). We searched the identified proteins against KOG classifications and the proteins were grouped into 25 classifications (Supplementary Table S2).

The PCA analysis was performed to evaluate the repeatability between samples and to assist in excluding outliers. PCA analysis showed that the sample M-2n-1 appeared to be a clear outlier (Supplementary Figure S3). Therefore, M-2n-1 was excluded from the subsequent analysis. After removing the outlier, the PCA showed that the repeatability of the samples was reliable.

### 3.3. Correlation Analysis of Transcriptome and Proteome in C. gigas

**Figure 2:**
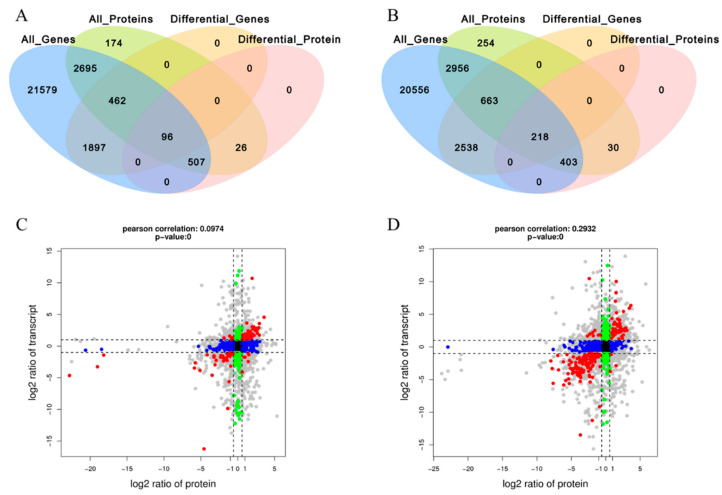
(**A**) The profile of differentially transcribed genes and expressed proteins between F-23nα (F-2n and F-3nα) and F-3nβ. (**B**) The profile of differentially transcribed genes and expressed proteins between M-23nα (M-2n and M-3nα) and F-3nβ. (**C**) Correlation analysis of the protein and mRNA expression between F-23nα and F-3nβ. (**D**) Correlation analysis of the protein and mRNA expression between M-23nα and M-3nβ. Each dot represents a gene/protein, black dots indicate non-differential proteins and genes, red dots indicate consistent or opposite trends in gene and protein changes, green dots indicate differentially expressed genes but non-differentially expressed proteins, and blue dots indicate non-differentially expressed genes but differentially expressed proteins. If the fold change is reached and the *p*-value is not reached, the dots are shown in gray.

According to association analysis of the proteome and transcriptome data, the sets of significantly differentially expressed genes and proteins were compared to determine gonad differential expression patterns. Results showed that of the 629 significantly differentially expressed proteins identified within the gonad proteome among female F-23nα (F-2n and F-3nα) and F-3nβ, expression of 96 corresponding genes were also significantly differentially expressed (Figure 2A), and the consistency between the two data sets was about 15.3%. Moreover, 218 of the 651 significantly differentially expressed proteins identified within the proteome corresponded with transcriptionally differentially expressed genes (Figure 2B) among male M-23nα (M-2n and M-3nα) and F-3nβ, which was generally 33.5% consistent. Therefore, transcriptomic and proteomic analysis showed that protein translation was partially coupled to gene transcription in *C. gigas* gonad.

The correlations of the transcriptome and proteome in gonadal tissues of C. gigas were analyzed. For females, significance analysis showed that 84 of the 96 mRNAs were consistent with the differential expression patterns of corresponding proteins (Supplementary Table S3), of which 50 were up-regulated and 34 were down-regulated in 3nβ (Figure 2C). In males, significance analysis showed that 207 of the 218 mRNAs were consistent with the differential expression patterns of corresponding proteins (Supplementary Table S4), of which 56 were up-regulated and 151 were down-regulated in 3nβ (Figure 2D). In addition, it indicated that genes with the same expression trend in transcriptome and proteome expressed proteins by a combination of transcriptional and translational mechanisms.

### 3.4. GO Enrichment Analysis between Fertile and Sterile Oysters

The 50 high expressed genes in female 3nβ compared to female 2n and 3nα were assigned to GO terms. The results showed that the most abundant high expressed genes encoded membrane (three genes) and cell periphery (three genes) (Supplementary Figure S4A). The molecular functional categories encoded by the high expressed genes were dominated by binding to ion (five genes) and cation (five genes) (Supplementary Figure S4B). The genes within the category of biological processes were classified as related to pyruvate metabolic process (two genes) and monocarboxylic acid metabolic process (two genes) (Supplementary Figure S4C). The 34 low expressed genes in female 3nβ showed that genes categorized as cellular components mainly encoded intracellular membrane-bounded organelle (nine genes) plus some with functions in the organelle part (seven genes), cytoplasm (six genes), and protein complex (six genes) (Supplementary Figure S4D). The molecular functional categories of genes were mainly attributable to binding with organic cyclic compound (ten genes), nucleic acid (eight genes), small molecule (eight genes), nucleoside (seven genes), and carbohydrate derivative (seven genes) (Supplementary Figure S4E). Many low expressed genes within the biological processes were assigned to cellular metabolic process (13 genes), macromolecule metabolic process (12 genes), and ‘cellular component organization or biogenesis’ (nine genes) (Supplementary Figure S4F).

The comparison of M-23nα (M-2n and M-3nα) and M-3nβ revealed that 56 high expressed genes in 3nβ males were assigned to GO terms. For results categorized as cellular components, the most high expressed proteins were intracellular part (six genes), organelle (five genes), and cytoplasm (four genes) (Supplementary Figure S5A). The genes within the category of molecular function were mainly attributable to binding with small molecule (three genes), purine nucleoside (two genes), and were involved in transmembrane transporter activity (two genes) (Supplementary Figure S5B), while a large number within the biological processes category were assigned to carbohydrate derivative metabolic process (two genes) (Supplementary Figure S5C). The results of this analysis demonstrated that the 151 low expressed genes in 3nβ males were mainly classified within the organelle part (21 genes), and non-membrane-bounded organelle (20 genes) (Supplementary Figure S5D). The molecular functional categories of the significantly low expressed genes were binding to organic cyclic compound (28 genes), small molecule (20 genes), carbohydrate derivative (18 genes), and nucleoside (17 genes) (Supplementary Figure S5E). Indeed, a significant proportion of the low expressed genes were presumably involved in biological processes including ‘cellular component organization or biogenesis’ (20 genes), and macromolecular complex subunit organization (13 genes) (Supplementary Figure S5F).

### 3.5. Key Pathways Related to Oyster Sterility Analysis by Proteome and Transcriptome

**Figure 3:**
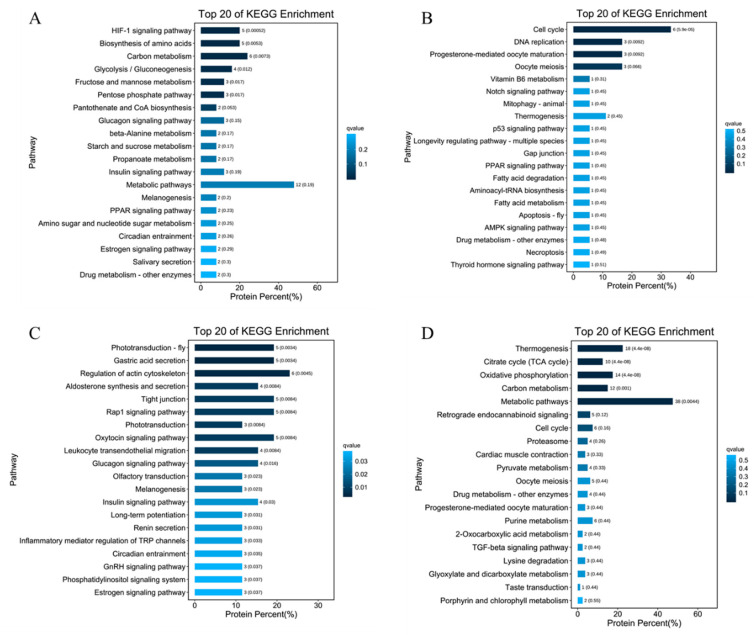
Enrichment of DEGs with a consistent pattern of DEPs between fertile oysters and sterile triploid oysters in KEGG pathways. (**A**) The upregulated genes in F-3nβ compared to F-23nα (F-2n and F-3nα). (**B**) The downregulated genes in F-3nβ compared to F-23nα (F-2n and F-3nα). (**C**) The upregulated genes in M-3nβ compared to M-23nα (M-2n and M-3nα). (**D**) The downregulated genes in M-3nβ compared to M-23nα (M-2n and M-3nα). The graphs are drawn by using the pathways of the top 20 sorted by q-value from small to large. The number of paths and the q-value are the values on the column. The ordinate is the pathway, and the abscissa is the percentage of the number of proteins in the pathway to the number of genes in all pathways.

To explore the underlying signaling pathways existing among the identified DEGs with a consistent pattern of DEPs between fertile oysters and sterile triploid oysters, KEGG pathway enrichment analysis was performed. For the upregulated genes in female 3nβ compared to female 2n and 3nα, the top 20 relevant pathways were illustrated in Figure 3A and the relevant genes were performed in Supplementary Table S5. The signaling pathways of interest included the biosynthesis of glycolysis/gluconeogenesis signaling pathway, insulin signaling pathway, and metabolic pathways. A significant proportion of the genes in these pathways are related to biosynthesis glycogen and fat such as Glys, Slc2a4, and GPAT4. For the downregulated genes in 3nβ females, the top 20 relevant pathways were displayed in Figure 3B and the relevant genes were performed in Supplementary Table S5. The signaling pathways of interest included the cell cycle, DNA replication, progesterone-mediated oocyte maturation, and oocyte meiosis pathways. Amongst the genes lowly expressed in 3nβ could be associated with DNA replication, cell cycle regulation, and spindle assembly such as CDK1, MCM3, MACM7, AURKA, and HDAC1.

For the upregulated genes in 3nβ males compared to male 2n and 3nα, the top 20 pathways were illustrated in Figure 3C and the relevant genes were performed in Supplementary Table S5. Here, we focused on the regulation of aldosterone synthesis and secretion, glucagon signaling, and insulin signaling pathways. Some genes were primarily involved in the synthesis of glycogen such as Slc2a4. For the downregulated genes in 3nβ males, the top 20 pathways were displayed in Figure 3D and the relevant genes were performed in Supplementary Table S5. The signaling pathways of interest included the cell cycle and TGF-beta signaling pathways. These genes were associated with spindle assembly such as MAD2L1.

### 3.6. Validation of Selected DEGs

**Figure 4:**
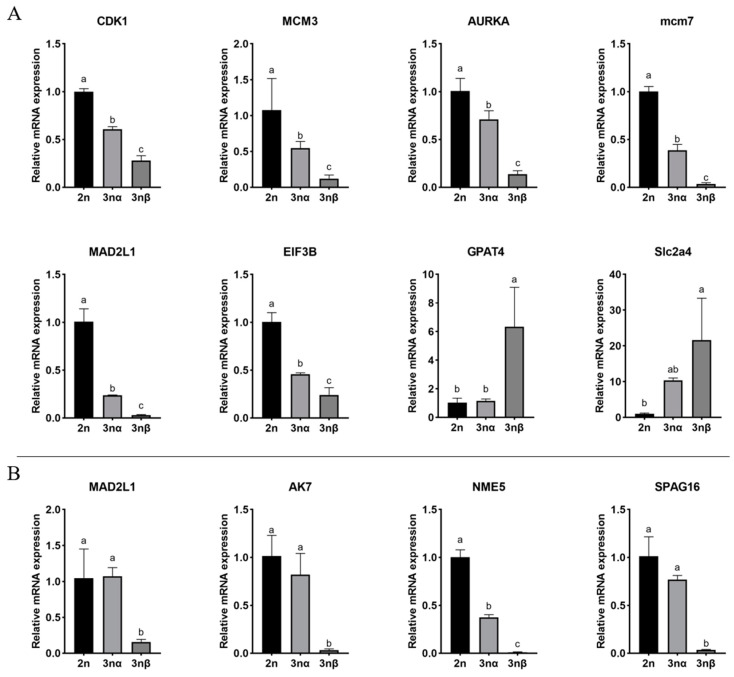
Validation of differentially expressed genes by qRT-PCR analysis. (**A**) DEGs between F-23nα (F-2n and F-3nα) and F-3nβ. (**B**) DEGs between M-23nα (M-2n and M-3nα) and M-3nβ. There are three replicates for each sample. Error bars indicated standard deviations of averages from three replicates. Significant differences were marked by different letters (a, b, c), *p* < 0.05.

To validate the results from the integration analysis, qRT-PCR was conducted. Eleven gonadal development-related DEGs with the same expression trend in transcriptome and proteomics were selected for mRNA expression verification by qRT-PCR. The results showed that eleven DEGs were consistent with the results of the transcriptomic analysis, suggesting that the transcriptomic data were reliable (Figure 4A,B). The geNorm calculates M values of candidate reference genes to represent the gene expression stability, where lower M values indicate more stable expression. The geNorm also calculated pairwise variation (Vn/n+1) to assess the number of reference genes for normalization. When Vn/n+1 is less than 0.15, n genes are enough for normalization. In the female gonadal comparison group, EF-1a, GAPDH, RS18, RO21, and RL7 were used for normalization (Supplementary Figure S6A,C). In the male gonadal comparison group, RS18 and RL7 were used for normalization (Supplementary Figure S6B,D).

## 4. Discussion

Gametogenesis is a very complex multifactorial process, and the expression of every related gene and protein involved is subject to fine regulation [36]. Deciphering the laws behind fecundity in triploid oysters plays a vital role in elucidating the gametogenesis mechanisms. Recently, transcriptomics and proteomics have been greatly productive in elucidating molecular mechanisms underlying the biological process. The transcriptome is a collection of transcriptional information for an organism. Transcriptomic analysis can elucidate and validate gene expression profiles. However, biological processes are driven by proteins and the evidence is mounting against messenger RNA (mRNA) being a reliable indicator of protein levels. Therefore, the combination of data from transcriptomic and proteomic analyses has been very beneficial in characterizing functional genes and proteins simultaneously [37]. This has helped to deduce the role of specific compounds and their regulatory pathways in the biological system. In our study, integrated proteomics and transcriptomics were performed to identify the candidate proteins/genes regulating gonadal development of triploids and to gain a deeper understanding of the molecular mechanisms of oyster gametogenesis. The sterile triploid oysters exhibited several significant differences in their molecular components and related signaling pathways compared to fertile oysters.

### 4.1. Downregulation of Mitotic Cell Cycle-Related Genes May Be Associated with Sterility of Triploid Females

The integrated proteomic and transcriptomic analysis presented here revealed that a large proportion of genes between female sterile triploids and fertile females were mainly enriched in the cell cycle, DNA replication, and oocyte meiosis pathways. Cell cycle progression is driven by the assembly of cell cycle-specific cyclins and their catalytic partner cyclin-dependent kinases (CDKs) [38]. During the S phase of the cell cycle, DNA replication is the main event, which doubles the number of chromosomes in cells. At the center of the molecular network controlling the cell cycle, CDKs promote cell cycle transition by phosphorylating specific downstream targets (including effectors of cell cycle events).

CDK1 was reported as the only essential cell cycle CDK in mammals [39]. CDK1 activity is required for DNA replication and meiotic recombination and acts early in meiosis [40]. In mammals, CDK1 is essential for promoting both oocyte meiotic resumption and mitotic cytokinesis [39,41]. The down-regulation of CDK1 expression may be related to the generation of sterility in triploid females. CDK1 would be probably linked with DNA replication control during the proliferation of oocytes in C. gigas. CDK1 lowly expressed in 3nβ females may affect mitotic oogonia, resulting in the failure of DNA replication and interfering with normal mitotic processes.

MCM3 and MCM7 are key molecules in the cell cycle and DNA replication [42], which are involved in the initiation of eukaryotic genome replication. MCM3 is a replicative helicase [43]. Acetylation of MCM3 triggers DNA replication and cell cycle progression [44], and cells lacking MCM3 fail to enter S phase and proliferate [45]. MCM7 is a key component of the pre-replication complex involved in the initiation of eukaryotic DNA replication. It plays an important role in initiating DNA replication during the G1 phase and extending DNA strands during the S phase. In 3nβ females, decreased expression of the two genes MCM3 and MCM7 may be associated with inhibition of DNA replication initiation during cell cycle progression, resulting in failure to initiate oocyte proliferation. In the previous study on C. gigas, the MCM3 was also found downregulated in 3nβ oysters [26]. These results suggest that MCM3 and MCM7 are likely to be involved in the proliferation of oocyte in *C. gigas*.

MAD2L1 and AURKA are the key proteins for spindle assembly. When they are abnormally expressed, they will cause chromosome mismatch and other genetic problems during mitosis [46]. MAD2L1 is a component of the mitotic spindle assembly checkpoint, and plays significant roles in protecting cells from abnormal chromosome segregation. Specifically, it can prevent late divisions from occurring until all chromosomes are properly aligned at the metaphase plate [47]. Among humans, inhibition of MAD2L1 expression inhibits the proliferation and migration of cancer cells [48,49]. AURKA is a cell cycle-regulated kinase involved in microtubule formation and/or spindle pole stabilization during chromosome segregation. AURKA has been implicated in the regulation of mitosis, cell cycle progression, and a key number of oncogenic signaling pathways in various malignancies [50]. The downregulation of MAD2L1 and AURKA indicates that abnormal spindle assembly may occur in 3nβ females, leading to abnormal chromosome segregation during cell division. These results suggest that MAD2L1 and AURKA may be closely associated with impaired oocyte production.

Moreover, HDAC1 can control cell proliferation and differentiation. It has been reported that silence of HDAC1 in cancer cells can either arrest at the G1 phase of the cell cycle or the G2/M transition, resulting in reduced number of mitotic cells, inhibited cell growth, and increased percentage of apoptotic cells [51,52,53]. In mice, loss of both HDAC1 and HDAC2 can induce apoptosis in mouse oocytes [54]. The downregulation of HDAC1 in 3nβ females indicates that cell proliferation and differentiation may be inhibited due to cell cycle arrest, and oocyte apoptosis is probably promoted. Indeed, oocyte apoptosis in gonadal tubules of triploid oysters was previously observed [23].

Overall, our results indicate that a primary cause of sterility in female triploid oysters may be the alteration in the expression levels of certain mitotic cell cycle-related genes/proteins. We speculate that DNA replication may be hindered, or cells may stagnate in the G1 phase or G2/M transition during the proliferation of oogonia in sterile females. For the cells that luckily pass through the interphase, they may also be due to spindle assembly abnormalities lead to chromosome segregation abnormalities during the division period, resulting in blocked oogenesis. However, the expression levels of mitotic cell cycle-related genes/proteins were upregulated in 3nα compared to 3nβ, suggesting that the oogonial proliferation in 3nα might be normal.

### 4.2. Downregulation of Genes Related to Cell Cycle and Sperm Motility in Sterile Male Triploids

In order to find out the factors that interfere gonad development in 3nβ male oysters, we searched for genes downregulated in 3nβ individuals. Pathway analysis indicated that the genes downregulated in sterile male oysters were mainly enriched in cell cycle and metabolic pathways, suggesting a stalled cell cycle and a change in metabolic activity occurred in spermatogenesis in 3nβ males.

CDK2 is a key regulator of cell cycle progression and plays an important role in G1/S transition and S-phase progression in mammals. However, it was reported that CDK2 is not necessary for the division of most mitotic cells. In mice, CDK2 is required for completion of prophase I of the meiotic cell cycle, and it appears to be essential for synaptonemal complex formation during the pachytene stage in male germ cells. Moreover, male germ cells will undergo apoptosis in the absence of CDK2 [55]. Our results illustrate that CDK2 was downregulated in 3nβ male oysters, suggesting that the first meiotic division (meiosis I) may be blocked during spermatogenesis.

Our results showed that MCM6 was downregulated in 3nβ males. As mentioned earlier, the MCM proteins are essential replication initiation factors. The most famous is a family of six structurally related proteins, and MCM6 is a member of this protein family. Such downregulation of MCM6 may cease the initiation of DNA replication during spermatogenesis, resulting in little cell proliferation.

In addition, MAD2L1, the spindle assembly-related gene, was also downregulated in 3nβ males, which indicated that the sterility in 3nβ males may be linked with abnormal spindle assembly.

Down-regulated proteins were also involved in spermatogenesis and sperm motility. NME5 gene encodes NME/NM23 family member 5, also known as non-transfer protein. It has been reported that NME5 may be related to spermatogenesis. In male Nme5^−/−^ knockout mice, late spermatogenesis arrest and flagella defects were observed [56]. SPAG16 protein is required for sperm flagellar motility function. The chimeric mice knocking out SPAG16L and SPAG16S transcripts have serious defects in spermatogenesis [57]. Such downregulation of NME5 and SPAG16 indicates that spermatogenesis in 3nβ males may have stopped and likely result in a reduced amount of flagellum motility. Previous study has demonstrated that the percentage of motile sperm and sperm swimming speed were lower in triploid C. gigas compared with diploid *C. gigas* [25].

In general, the disruption of mitosis or meiosis may explain the blocking of spermatogenesis in 3nβ males. The sperm flagellum is the key factor affecting sperm motility. The male sterile triploids do produce very few gametes, but these gametes may have poor sperm motility due to flagella defects. However, the expression levels of meiosis-related and sperm flagellum-related genes/proteins were upregulated in 3nα compared to 3nβ, which probably underlies the 3nα oysters can generate normal sperm.

### 4.3. Sterile Triploid Oysters Exhibited Increased Biosynthesis of Glycogen and Fat

In our study, we observed an increase in gene/protein abundance related to the biosynthesis of glycogen and fat, including Glys, Slc2a4, and GPAT4 in 3nβ female oysters compared to female 2n and 3nα. Glys was enhanced in female sterile triploid oysters, which implied increased biosynthesis of glycogen in the female 3nβ individuals. It has also been reported that in C. gigas, Glys was highly expressed in individuals with high glycogen content, and participated in glycogen biosynthesis [58]. Slc2a4 can regulate glucose transport. Increased expression of Slc2a4 promotes glucose transport to fat and muscle to reduce glucose levels and this process is usually regulated by insulin [59]. The high expression level of Slc2a4 involved in glucose transport may participate in the process of glucose conversion to glycogen in 3nβ individuals, which increases glycogen content in 3nβ female oysters. GPAT catalyzes the first step during de novo synthesis of glycerolipids. GPAT4 can promote the differentiation of fat cells. GPAT catalyzes the first step during de novo synthesis of glycerolipids and insulin also plays a critical role in this process. Targeted knockdown of GPAT3 significantly decreased GPAT activity and impaired adipocyte differentiation in 3T3-L1 cells of mice [60]. In short, a high level of GPAT4 expression may increase fat content in the gonad of 3nβ females. In our study, the insulin signaling pathway was highly expressed in sterile triploid oysters, which may be related to the regulation of insulin expression in both Slc2a4 and GPAT4. Genes enriched in the insulin pathway may further influence glycogen synthesis by regulating insulin expression.

Curiously, of the three genes mentioned above, only Slc2a4 was significantly differentially expressed in the male comparison group M-23nα (M-2n and M-3nα) and M-3nβ, while the expression of the other two genes Glys and GPAT4 did not differ. Fat has an important impact on the gonadal development of shellfish. It is an energy substance for maintaining gonadal development and an important nutrient component in oocytes, which has been reported previously [61]. The high expression of GPAT4 in 3nβ females may be associated with a very low number of oocytes.

Overall, these glycogen synthesis-related genes were significantly upregulated in sterile triploids, which was consistent with previous observations that 3nβ was higher than 2n and 3nα at the same gonadal development stage [62]. Our findings suggest highly reduced gonad development may stimulate the synthesis of glycogen and energy conservation in gonad tissue.

## 5. Conclusions

In this study, we first performed integrated proteomics and transcriptomics in the gonad among diploid and triploid Pacific oysters and excavated genes/proteins related to the gonadal sterility of triploids.

The findings provide new insights into the regulatory mechanism responsible for sterility in triploid oysters. By combing proteomics and transcriptomics, we suggest that the molecular mechanism of female sterility in triploids was mainly associated with driven cell cycle arrest caused by downregulation of cell cycle-related genes, and the sterility of triploid male oysters was linked with cell division hindrance and decreased sperm motility. The past study has demonstrated that the apoptotic cells in gonads are due to the upregulation of apoptosis-associated genes. To the best of our knowledge, the accurate entry of cells into the division cycle is a prerequisite for maintaining normal cell proliferation. We consider that downregulated cell cycle-related genes in gonad can cause proliferating cells to stagnate at a certain point in the cell cycle, and the cells could not complete the normal cell cycle to achieve cell division, increasing the percentage of apoptotic cells.

We also consider that sterile triploids increase the partitioning of energy for biosynthesis of glycogen, due to the blocked gametogenesis. These findings improve our understanding of molecular mechanisms of sterility in triploid oysters and could promote research into the molecular breeding of oysters.

## Figures and Tables

**Table 1:** Primer nucleotide sequences used in this study.

Name	Sequences (5′ to 3′)	Product Size (bp)	Amplification Efficiency (%)
CDK1_F	ACTGGCAGACTTTGGATTGG	85	93.7
CDK1_R	GGCTCTGTACCATAGCGTCA		
MCM3_F	ATGACAAGTGCCGTCTCGTT	198	103.6
MCM3_R	CACCAAAGCTGCCCTCAAAC		
GPAT4_F	CACACGTCTCCACTGGATGT	93	109.6
GPAT4_R	CATGACCAGTCCCAGGAACC		
Slc2a4_F	TGGGCGTGTCCAAACTCT	154	101.5
Slc2a4_R	CTGGCTTCTTCCTCTTCATTCT		
MAD2L1_F	TCCGTCACATTCCTACCC	140	95.5
MAD2L1_R	CGCAGTCTCACTTCCTCC		
EIF3B_F	ATGATGTCGGAAACCTGC	147	95.6
EIF3B_R	CAGTCCACCTTGCTCTTT		
AURKA_F	AGTTCTTTCATCCGGGCACT	147	104.2
AURKA_R	TGGCTCTTTCCTGCTTGGTT		
mcm7_F	GATGGAGGGTGACAGAACCG	101	95.9
mcm7_R	TGGAGACCCTAGCGTTGAGA		
SPAG16_F	TGCCCTAGCAACATCTCAGA	189	92
SPAG16_R	ACCACTCCGATGTCCTCCTC		
NME5_F	GCCATAGTGATAGCAAGAGACCA	130	91.3
NME5_R	TGCGCTGATCATCTGTTCCA		
AK7_F	AGCCAAGACGAAGAGAAGCC	156	92.4
AK7_R	GCTGGTGGTTGACCTAGCAT		
EF-1α_F	AGTCACCAAGGCTGCACAGAAAG	200	100.2
EF-1α_R	TCCGACGTATTTCTTTGCGATGT		
GAPDH_F	TTCTCTTGCCCCTCTTGC	127	99.9
GAPDH_R	CGCCCAATCCTTGTTGCTT		
RO21_F	AATGCCAGGCTAACAGACCACA	100	93.2
RO21_R	TTGGATTTCTGAGATTCCGATCTTC		
RS18_F	GCCATCAAGGGTATCGGTAGAC	168	99.1
RS18_R	CTGCCTGTTAAGGAACCAGTCAG		
RL7_F	TCCCAAGCCAAGGAAGGTTATGC	242	101.7
RL7_R	CAAAGCGTCCAAGGTGTTTCTCAA		

## Data Availability

The data presented in this study are available on request from the corresponding author.

## Supplementary Materials

The following are available online at https://www.mdpi.com/article/10.3390/cells10102668/s1, Figure S1: Flow-cytometric histogram of female diploid (A), female fertile triploid (B), female sterile triploid (C), male diploid (D), male fertile triploid (E), and male sterile triploid (F), Figure S2: (A) Statistical analysis for protein identification. (B) Annotation of proteins in three public databases (GO, KEGG and KOG), Figure S3: The principal component analysis (PCA) of expression patterns of proteins from *Crassostrea gigas*, a indicates α; b indicates β, Figure S4: The top 20 of GO enrichment of significantly high expressed genes in 3nβ females compared to 2n and 3nα females are subjected to three categories: (A) cellular component, (B) molecular function and (C) biological process. The significantly low expressed genes in 3nβ females are categorized into (D) cellular component, (E) molecular function, as well as (F) biological process, Figure S5: The top 20 of GO enrichment of significantly high expressed genes in 3nβ males compared to 2n and 3nα males are subjected to three categories: (A) cellular component, (B) molecular function and (C) biological process. The significantly low expressed genes in 3nβ males are categorized into (D) cellular component, (E) molecular function, as well as (F) biological process, Figure S6: Average expression stability values of the candidate reference genes under fertile and infertile female gonads (A), and fertile and infertile male gonads (B) analyzed by geNorm. The number of reference genes calculated by geNorm in fertile and infertile female gonads (C), and fertile and infertile male gonads (D), Table S1: The whole proteins in gonads of *C. gigas*, Table S2: Annotated KOG pathways in gonadal proteome data, Table S3: Genes with a consistent pattern of differential expression between transcriptome and proteome of F-23nα (F-2n and F-3nα) and F-3nβ, Table S4: Genes with a consistent pattern of differential expression between transcriptome and proteome of M-23nα (M-2n and M-3nα) and M-3nβ, Table S5: Genes from the top 20 enriched KEGG pathways in the comparison of F-23nα (F-2n and F-3nα) and F-3nβ, Table S6: Genes from the top 20 enriched KEGG pathways in the comparison of M-23nα (M-2n and M-3nα) and M-3nβ.
