# Supplementary material for: Integrated Proteomic and Transcriptomic Analysis of Gonads Reveal Disruption of Germ Cell Proliferation and Division, and Energy Storage in Glycogen in Sterile Triploid Pacific Oysters (Crassostrea gigas)
Source: Cells. 2021 Oct 5;10(10):2668. doi: 10.3390/cells10102668 (PMC8534442; doi:10.3390/cells10102668)
Supplement: Supplementary file 1 [file cells-10-02668-s001.zip › Supplementary Figures.pdf]

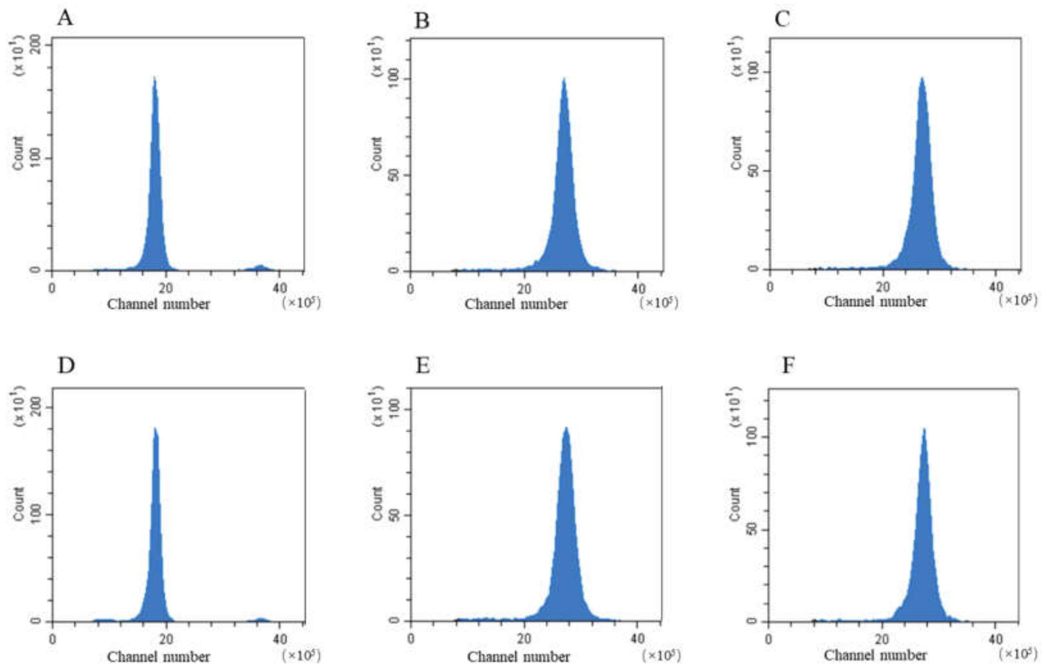

Figure S1. Flow-cytometric histogram of female diploid (A), female fertile triploid (B), female sterile triploid (C), male diploid (D), male fertile triploid (E), and male sterile triploid (F).

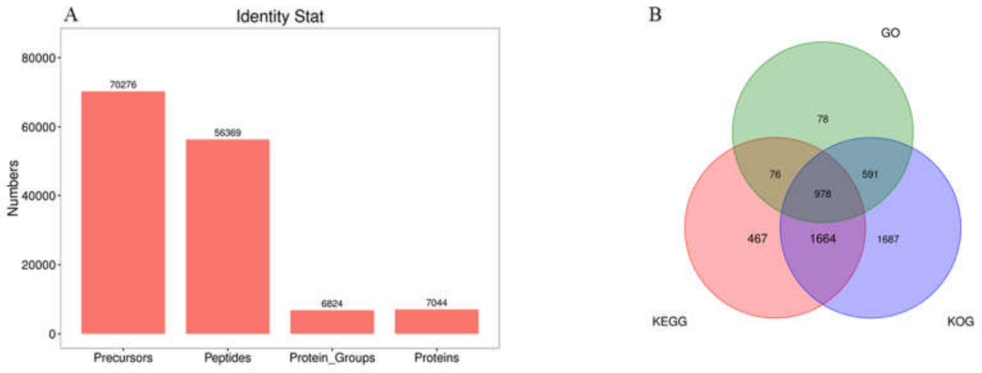

Figure S2. (A) Statistical analysis for protein identification. (B) Annotation of proteins in three public databases (GO, KEGG and KOG).

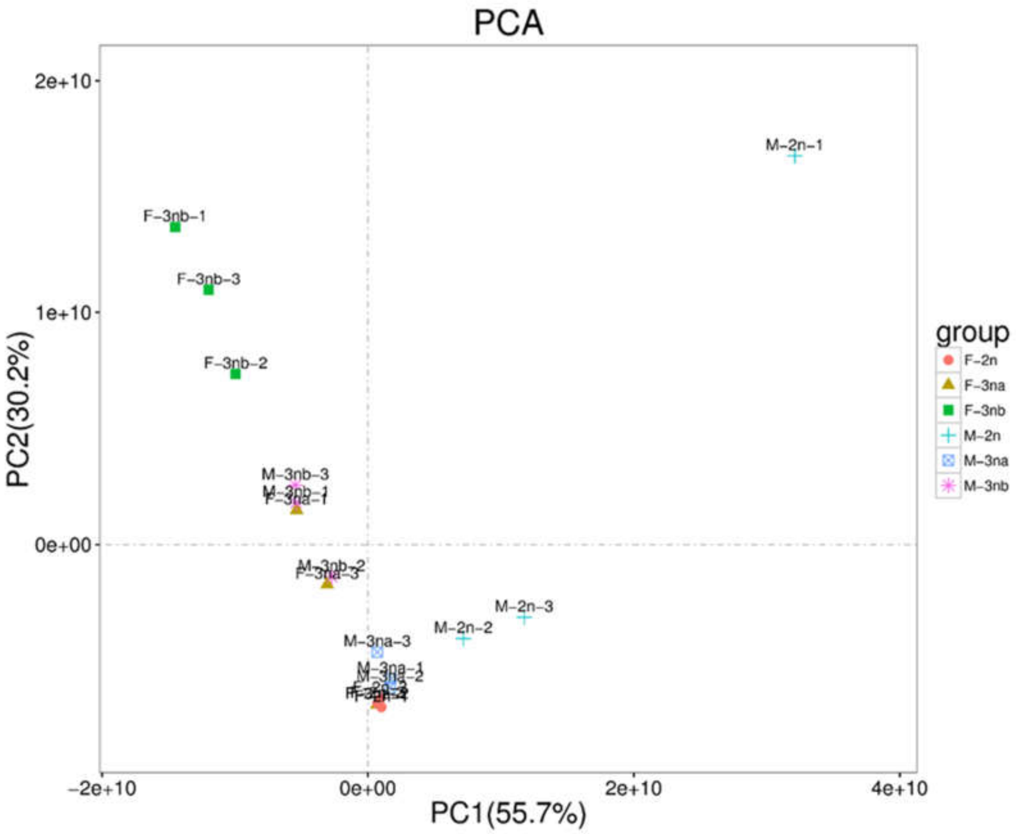

Figure S3. The principal component analysis (PCA) of expression patterns of proteins from *Crassostrea gigas*, a indicates α; b indicates β.

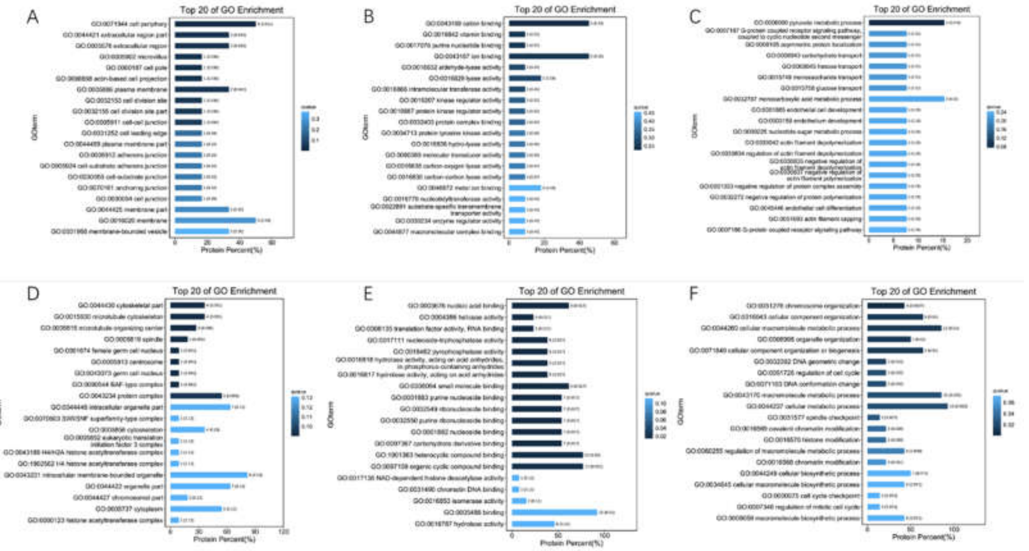

Figure S4. The top 20 of GO enrichment of significantly high expressed genes in 3nβ females compared to 2n and 3nα females are subjected to three categories: (A) cellular component, (B) molecular function and (C) biological process. The significantly low expressed genes in 3nβ females are categorized into (D) cellular component, (E) molecular function, as well as (F) biological process.

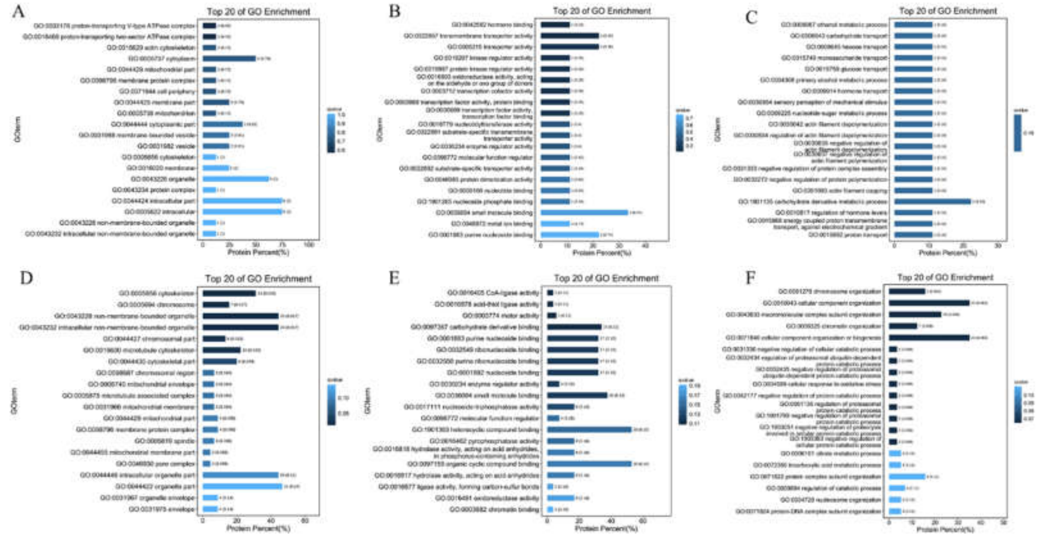

Figure S5. The top 20 of GO enrichment of significantly high expressed genes in 3nβ males compared to 2n and 3nα males are subjected to three categories: (A) cellular component, (B) molecular function and (C) biological process. The significantly low expressed genes in 3nβ males are categorized into (D) cellular component, (E) molecular function, as well as (F) biological process.

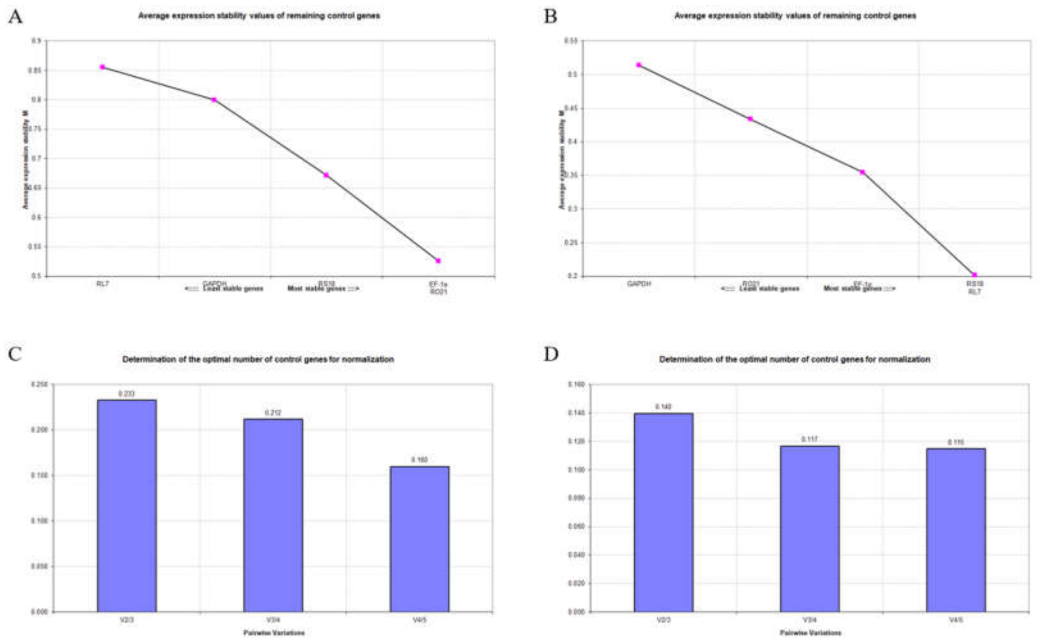

Figure S6. Average expression stability values of the candidate reference genes under fertile and infertile female gonads (A), and fertile and infertile male gonads (B) analyzed by geNorm. The number of reference genes calculated by geNorm in fertile and infertile female gonads (C), and fertile and infertile male gonads (D).
