# Supplementary material for: Integrated Proteomic and Transcriptomic Analysis of Gonads Reveal Disruption of Germ Cell Proliferation and Division, and Energy Storage in Glycogen in Sterile Triploid Pacific Oysters (Crassostrea gigas)
Source: Cells. 2021 Oct 5;10(10):2668. doi: 10.3390/cells10102668 (PMC8534442; doi:10.3390/cells10102668)
Supplement: Supplementary file 1 [file cells-10-02668-s001.zip › Supplementary Table S5..pdf]

Table S4. Genes from the top 20 enriched KEGG pathways in the comparison of F-23nα (F-2n and F-3nα) and F-3nβ

No.	log2FC[1] (mRNA)	log2FC[1] (protein)	Symbol	Description
Upregulated				
1	1.058	1.233	PGK1	LOW QUALITY PROTEIN: phosphoglycerate kinase 1-like
2	1.618	1.371	FBPA	fructose-bisphosphate aldolase-like
3	1.216	1.244	FBPA	Fructose-bisphosphate aldolase
4	1.979	1.356	ENO	enolase-like isoform X2
5	2.318	1.728	Slc2a4	solute carrier family 2, facilitated glucose transporter member 1-like
6	1.468	1.309	Os02g0714600	ribose-phosphate pyrophosphokinase 4-like
7	1.442	1.021	MUT	methylmalonyl-CoA mutase, mitochondrial-like
8	1.176	0.687	MPI	mannose-6-phosphate isomerase-like
9	2.426	1.407	DPYS	dihydropyrimidinase-like isoform X1
10	2.225	1.696	Oant_2987	uncharacterized protein LOC105328670 isoform X1
11	1.410	1.606	GlyS	glycogen [starch] synthase-like isoform X1
12	2.217	1.625	CPK19	Calcium-dependent protein kinase isoform 2
13	1.618	1.998	UGP2	UTP--glucose-1-phosphate uridylyltransferase-like isoform X3
14	1.509	1.681	GRE2	LOW QUALITY PROTEIN: putative uncharacterized oxidoreductase YDR541C
15	1.479	1.881	PRKAR1A	cAMP-dependent protein kinase regulatory subunit-like isoform X2
16	1.256	1.676	GPAT4	glycerol-3-phosphate acyltransferase 3-like
17	1.244	0.645	SCGOA	guanine nucleotide-binding protein G(o) subunit alpha isoform X1
18	1.702	2.242	ILK	integrin-linked protein kinase-like isoform X1
19	2.042	2.667	--	perilipin-2-like isoform X2
Downregulated				
1	-1.557	-1.133	cdk1	cyclin dependent kinase 1
2	-2.949	-3.071	mcm7	DNA replication licensing factor mcm7-like
3	-1.755	-2.322	MAD2L1	mitotic spindle assembly checkpoint protein MAD2A-like isoform X1
4	-1.533	-3.212	MCM3	zygotic DNA replication licensing factor mcm3-like
5	-1.758	-3.212	zmcm3	zygotic DNA replication licensing factor mcm3-like
6	-1.140	-1.090	HDAC1	hypothetical protein LOTGIDRAFT_178649
7	-1.821	-2.018	AURKA	aurora kinase A-like
8	-1.149	-0.596	SNZERR	pyridoxal 5'-phosphate synthase subunit SNZERR-like
9	-3.260	-19.021	USP33	ubiquitin carboxyl-terminal hydrolase 7-like
10	-1.493	-2.269	cpt2	carnitine O-palmitoyltransferase 2, mitochondrial-like
11	-1.031	-0.783	Actl6a	actin-like protein 6B
12	-1.640	-1.048	Aars	-
13	-1.204	-1.466	AIFM1	apoptosis-inducing factor 1, mitochondrial-like
14	-9.858	-1.350	eef-2	hypothetical protein CGI_10017178
15	-1.433	-1.304	NME7	nucleoside diphosphate kinase 7-like

[1] log2FC: 3nβ/23nα.
