# Supplementary material for: Integrated Proteomic and Transcriptomic Analysis of Gonads Reveal Disruption of Germ Cell Proliferation and Division, and Energy Storage in Glycogen in Sterile Triploid Pacific Oysters (Crassostrea gigas)
Source: Cells. 2021 Oct 5;10(10):2668. doi: 10.3390/cells10102668 (PMC8534442; doi:10.3390/cells10102668)
Supplement: Supplementary file 1 [file cells-10-02668-s001.zip › Supplementary Table S6..pdf]

Table S5. Genes from the top 20 enriched KEGG pathways in the comparison of M-23nα (M-2n and M-3nα) and M-3nβ

No.	log2FC[1] (mRNA)	log2FC[1] (protein)	Symbol	Description
Upregulated				
1	2.802	2.193	mlc-3	myosin, essential light chain, adductor muscle-like
2	3.177	1.747	SPEC1	caltractin-like
3	4.102	1.936	CPK19	Calcium-dependent protein kinase isoform 2
4	8.299	1.501	ACTG1	actin-like
5	3.449	1.160	act-2b	actin-2-like
6	2.801	1.016	Actn	alpha-actinin, sarcomeric-like isoform X1
7	2.476	2.120	COF1	cofilin-like
8	3.017	2.049	gelsolin	gelsolin-like protein 2
9	1.877	1.090	Myl9	myosin regulatory light polypeptide 9-like
10	2.248	1.672	SLC9A3R1	Na(+)/H(+) exchange regulatory cofactor NHE-RF2-like isoform X1
11	2.298	1.821	STARD5	stAR-related lipid transfer protein 5-like isoform X1
12	2.606	1.132	Slc2a4	solute carrier family 2, facilitated glucose transporter member 1-like
13	2.222	1.574	PRKAR1A	cAMP-dependent protein kinase regulatory subunit-like isoform X2
Downregulated				
1	-1.102	-2.480	sdha-a	succinate dehydrogenase [ubiquinone] flavoprotein subunit, mitochondrial-like
2	-1.039	-2.107	UBCRBP	cytochrome b-c1 complex subunit 7-like
3	-1.127	-1.868	--	ATP synthase F(0) complex subunit B1, mitochondrial-like
4	-1.252	-1.834	ATP5F1A	ATP synthase subunit alpha, mitochondrial
5	-1.190	-0.872	ATPsynO	ATP synthase subunit O, mitochondrial-like
6	-1.121	-1.704	--	mitochondrial ATP synthase-like protein, partial
7	-1.263	-2.514	--	probable ATP synthase subunit g 1, mitochondrial
8	-1.456	-2.210	COX4I1	cytochrome c oxidase subunit 4 isoform 1, mitochondrial-like
9	-1.285	-2.044	Cox5a	cytochrome c oxidase subunit 5A, mitochondrial-like
10	-1.607	-3.833	NDUFS1	NADH-ubiquinone oxidoreductase 75 kDa subunit, mitochondrial-like
11	-1.483	-1.962	NDUFS8	NADH-ubiquinone oxidoreductase subunit 8-like
12	-1.298	-3.067	NDUFV1	NADH dehydrogenase [ubiquinone] flavoprotein 1, mitochondrial-like
13	-1.122	-2.495	--	NADH dehydrogenase [ubiquinone] 1 alpha subcomplex subunit 8-like
14	-1.259	-2.379	Ndufa9	NADH dehydrogenase [ubiquinone] 1 alpha subcomplex subunit 9, mitochondrial-like
15	-3.899	-4.577	actbc	actin, cytoplasmic-like
16	-1.918	-2.739	cpt2	carnitine O-palmitoyltransferase 2, mitochondrial-like
17	-5.613	-5.298	Npr1	atrial natriuretic peptide receptor 1-like
18	-1.953	-2.034	Slc25a20	mitochondrial carnitine/acylcarnitine carrier protein-like
19	-3.520	-6.853	--	putative malate dehydrogenase 1B
20	-2.317	-3.739	Idh3a	isocitrate dehydrogenase [NAD] subunit alpha, mitochondrial-like
21	-1.266	-2.913	PDHB	pyruvate dehydrogenase E1 component subunit beta, mitochondrial-like
22	-2.612	-3.371	ogdh	2-oxoglutarate dehydrogenase, mitochondrial-like isoform X5
23	-1.585	-1.421	Dlat	dihydrolipoyllysine-residue acetyltransferase component of pyruvate dehydrogenase complex, mitochondrial-like
24	-1.204	-1.335	Dlst	dihydrolipoyllysine-residue succinyltransferase component of 2-oxoglutarate dehydrogenase complex, mitochondrial-like
25	-1.168	-1.640	CS	citrate synthase, mitochondrial-like isoform X1
26	-1.429	-1.876	Suclg1	succinyl-CoA ligase subunit alpha, mitochondrial-like isoform X3
27	-1.877	-1.562	Sucla2	succinyl-CoA ligase [ADP-forming] subunit beta, mitochondrial-like
28	-1.131	-1.512	ME1	NADP-dependent malic enzyme-like
29	-9.145	-0.902	GLDC	glycine dehydrogenase (decarboxylating), mitochondrial-like isoform X2

30	-5.583	-7.642	--	uncharacterized protein LOC105327695
31	-5.583	-7.642	--	uncharacterized protein LOC105327695
32	-4.255	-4.125	NME5	nucleoside diphosphate kinase homolog 5-like
33	-5.285	-3.702	--	uncharacterized protein LOC101859880
34	-1.314	-6.973	Smp_194770	taurocyamine kinase-like
35	-4.470	-3.673	AK7	adenylate kinase 7-like
36	-3.289	-4.679	NME7	nucleoside diphosphate kinase 7-like
37	-5.150	-5.800	--	heme-binding protein 2-like isoform X1
38	-3.308	-4.152	Pacrg	parkin coregulated gene protein homolog isoform X1
39	-1.693	-2.036	MYCBP	C-Myc-binding protein
40	-1.934	-1.900	HADHB	trifunctional enzyme subunit beta, mitochondrial-like
41	-1.056	-1.982	hsdl2	hydroxysteroid dehydrogenase-like protein 2 isoform X2
42	-3.639	-4.781	SPAG16	sperm-associated antigen 16 protein-like isoform X2
43	-2.249	-3.385	CDK2	cyclin-dependent kinase 2-like
44	-1.254	-2.267	MAD2L1	mitotic spindle assembly checkpoint protein MAD2A-like isoform X1
45	-1.910	-2.267	MAD2L1	mitotic spindle assembly checkpoint protein MAD2A-like isoform X1
46	-1.597	-2.243	Mcm6	hypothetical protein LOTGIDRAFT_206985
47	-1.136	-1.821	SKP1	S-phase kinase-associated protein 1
48	-1.075	-1.338	PPP2CB	serine/threonine-protein phosphatase 2A catalytic subunit beta isoform isoform X1
49	-1.264	-2.041	PCNA	proliferating cell nuclear antigen-like
50	-1.321	-0.870	PSMB7	proteasome subunit beta type-7-like
51	-1.497	-2.173	PSMD2	26S proteasome non-ATPase regulatory subunit 2-like
52	-1.239	-0.808	PSMC4	26S protease regulatory subunit 6B isoform X2
53	-1.036	-1.280	PSMC6	26S protease regulatory subunit 10B
54	-2.737	-5.321	Pde1c	-
55	-1.266	-1.711	kmt5a	N-lysine methyltransferase SETD8-like

[1] log2FC: 3nβ/23nα.
